# The Rev. Mr. Jenner, on Vaccine

**Published:** 1801-05

**Authors:** G. C. Jenner

**Affiliations:** No. 13, Bond Street


### Medical and Phyſical Journal.

VOL. V.] MAY, 1801. [NO. XXVII.

### *To the Editors of the Medical and Phyſical Journal.*

GENTLEMEN,

IT will perhaps be thought unneceſſary at this period, when the world is already furniſhed with ſuch abundant evidence of the ſafety of the Vaccine Inoculation, and its efficacy in protecting the human conſtitution againſt the Small-pox, to advance any thing further on the ſubject; but having lately ſeen in the public papers ſome letters, tending to excite alarm among thoſe who have adopted the new ſyſtem of inoculation, I could not refrain from offering a few words, with a hope that they may allay any unpleaſant ſenſations ariſing from this ſource.

Since the month of December, 1799, I have inoculated nearly three thouſand perſons for the Cow-pox, a very great number of whom have ſince been expoſed to the contagion of the Small-pox, in every way that can be imagined, without its producing the ſmalleſt effect. To place this in a point of view, at once ſtriking and within the reach of inquiry, I ſhall mention an inoculation which took place at a village in Wiltſhire, *Burbage*, near the ſeat of the Earl of Aileſbury. Being there on a viſit laſt ſpring, I gave a general invitation to people of all deſcriptions to come and be inoculated. My offer was accepted by men, women and children, to the amount of ſix hundred, who went through the Cow-pox in the uſual mild way. Some months afterwards the village was viſited by the Small-pox, which made dreadful havoc among many of thoſe who did not avail themſelves of my invitation, while thoſe who accepted it remained ſecure, although there was a general intercourſe between them during the whole period. Facts equally ſtrong have been tranſmitted to me by a letter from my very particular friend, the Rev. Mr. Clinch, of Trinity, in Newfoundland, of which the following is an extract:

" The threads you ſent me produced the deſired effect, which proved a happy circumſtance for this harbour. After inoculating my own family, I availed myſelf of the opportunity, whilſt the Small-pox was making its ravages at St. John's, of viſiting that place. Encouraged by your reprefentation, and in order to eftablifh the fact of the Cow-pox being an abfolute preventive of the Small-pox, I put my nephew (Jofeph Hart) to the moft rigid teft, by inoculating him with active variolous matter, and expofing him to a contagious atmofphere, but without its producing in either inftance the fmalleft effect on the fyftem. This fingle cafe excited the aftonifhment of every perfon within whofe knowledge it came; and moft of thofe who had not previoufly gone through the Small-pox, were eager to fhield themfelves againft that dreadful malady by adopting the Vaccine Inoculation. Juft before my arrival at St. John's, a woman was inoculated for the Small-pox, and, four days afterwards, her infant at the breaft, with the Vaccine. Both went through the refpective difeafes in the ufual way, and perfectly diftinct from each other, although the mother continued to fuckle her child the whole time. Shortly after my return to this place, the Small-pox was imported in a veffel from Quebec; one of her crew died of it. Fortunately for the inhabitants of Trinity, moft of them had been inoculated with the Cow-pox, and were thereby prepared to refift the influence of the Small-pox. Several of my Cow-pox patients attended this man during his illnefs, but efcaped the infection of his difeafe.

" It gives me great pleafure to fay that the Jennerian Inoculation is extending itfelf faft over this ifland; but one thing I lament is, the great probability of many errors being committed by perfons ignorant of the difeafe, and inexperienced in the practice of inoculation; for the people of Newfoundland are indifcriminately inoculating each other, and I fear fome of them may either not receive the infection at all, or by taking the virus at too late a period (a circumftance you fo particularly cautioned me againft) create a fpurious difeafe; and thus, under an ideal fecurity, expofe themfelves to variolous contagion, when, in fact, they have never had the fpecific preventive. I muft add that we are much indebted to the zeal and exertions of Mr. Macurdy, furgeon, of St. John's, for the rapid progrefs the Vaccine Inoculation has made in Newfoundland. Mr. Macurdy, I underftand, received his virus from Dr Jenner, through the medium of our worthy and refpected Governor, Admiral Pole."

Having in my own extenfive inoculation feen the vaccine puftule in all the varieties it affumes, I confider it as a duty I owe to humanity and fociety to anfwer any enquiries that may be made on the fubject; and if the refult of my information fhould contribute to relieve the anxiety of a parent for the fafety of a child, or remove the pain of fufpence, I fhall feel myfelf highly gratified. I am, &c.

G. C. JENNER.

*No.* 13, *Bond Street,*

